# Reduction in Rheumatoid Factor and Matrix Metalloproteinase-3 With Successful Glucocorticoid Withdrawal After Initiation of Pemafibrate in a Patient With Rheumatoid Arthritis and Hypertriglyceridemia: A Case Report

**DOI:** 10.7759/cureus.116509

**Published:** 2026-09-18

**Authors:** Kimihiro Suzuki

**Affiliations:** 1 Rheumatology, Suzuhiro Clinic, Saitama, JPN

**Keywords:** glucocorticoid-sparing effect, glucocorticoid withdrawal, hypertriglyceridemia, matrix metalloproteinase-3, nuclear factor-κb, pemafibrate, rheumatoid arthritis, rheumatoid factor, selective pparα modulator

## Abstract

Pemafibrate is a selective peroxisome proliferator-activated receptor α (PPARα) modulator (SPPARMα) used for hypertriglyceridemia. PPARα activation also suppresses nuclear factor-κB (NF-κB) signaling, a pathway central to synovial inflammation and joint destruction in rheumatoid arthritis (RA). Whether pemafibrate affects RA-related biomarkers in humans is unknown.

A 51-year-old woman (height: 164 cm, body weight: 71 kg, body mass index: 26.4 kg/m²) with RA treated with methotrexate, bucillamine, iguratimod, and low-dose prednisolone (0.7 mg/day) had hypertriglyceridemia with coexisting metabolic dysfunction-associated steatotic liver disease (MASLD). Pemafibrate was started at 0.2 mg/day and increased to 0.4 mg/day after 12 weeks, while the disease-modifying antirheumatic drug (DMARD) regimen was kept unchanged throughout. Over the 20 months following the initiation of pemafibrate, rheumatoid factor (RF) and matrix metalloproteinase-3 (MMP-3) declined, and C-reactive protein (CRP) showed an overall decrease with a transient rise at month 6; prednisolone was tapered and discontinued after 17 months. The 28-joint Disease Activity Score based on C-reactive protein (CRP; DAS28-CRP), already in the remission range at baseline (1.9), remained below the remission threshold throughout. Serum triglycerides fell from 184 mg/dL to 88 mg/dL, and hepatic transaminases (aspartate aminotransferase (AST) and alanine aminotransferase (ALT)) showed a transient early increase followed by a return toward baseline, without meeting criteria for drug-induced liver injury. No adverse events attributable to pemafibrate were observed.

In this patient, the introduction of pemafibrate was temporally associated with reductions in RA-related biomarkers and with successful glucocorticoid withdrawal, without any intensification of DMARD therapy. Because the baseline prednisolone dose was already low (0.7 mg/day), this withdrawal should be interpreted cautiously and should not be regarded as strong evidence of a glucocorticoid-sparing effect. Causality cannot be established from a single case; nevertheless, together with earlier clinical reports on fenofibrate, animal studies showing anti-arthritic effects of fenofibrate and bezafibrate, and preclinical evidence of PPARα-mediated NF-κB inhibition, this observation suggests that PPARα agonists merit further investigation as adjunctive agents in RA, particularly in patients with coexisting dyslipidemia or steatotic liver disease.

## Introduction

Pemafibrate is a selective peroxisome proliferator-activated receptor α (PPARα) modulator (SPPARMα), a class distinguished from conventional fibrates by enhanced PPARα binding potency and subtype selectivity. Beyond its lipid-modifying effects, pemafibrate also exerts anti-inflammatory activity through PPARα activation, at least in part by negatively regulating nuclear factor-κB (NF-κB), a master regulator of inflammation, thereby suppressing the expression of downstream mediators such as tumor necrosis factor-α (TNF-α), interleukin-1β, and intercellular adhesion molecule-1 [1].

In rheumatoid arthritis (RA), NF-κB and TNF-α play a central role in synovial inflammation and joint destruction [2,3]. Given that pemafibrate can attenuate this same inflammatory axis, it is plausible that PPARα activation exerts a beneficial effect on RA disease activity. Here, we report a case in which, following the introduction of pemafibrate, rheumatoid factor (RF) and matrix metalloproteinase-3 (MMP-3) levels decreased and concomitant low-dose glucocorticoid therapy could be discontinued, while the 28-joint Disease Activity Score based on C-reactive protein (CRP; DAS28-CRP [4,5]) remained below the remission threshold throughout the course.

## Case presentation

A 51-year-old Japanese woman had been followed at our clinic for seropositive RA diagnosed 11 years before year X (X-11) according to the 2010 American College of Rheumatology/European League Against Rheumatism classification criteria [6,7]. At diagnosis, RF was 20 IU/mL and anti-cyclic citrullinated peptide antibody was >100 U/mL, with several involved joints (proximal interphalangeal (PIP) joints: right II, III, and left III; metacarpophalangeal (MP) joints: right II, III). Her comorbidities were hypertriglyceridemia and metabolic dysfunction-associated steatotic liver disease (MASLD), the latter diagnosed by abdominal ultrasonography. She had no history of alcohol excess.

At the time pemafibrate was initiated in X.Y (year X, month Y), her height was 164 cm and her body weight was 71 kg, corresponding to a body mass index of 26.4 kg/m². Her antirheumatic regimen consisted of methotrexate 8 mg/week, bucillamine 200 mg/day, iguratimod 50 mg/day, and prednisolone 0.7 mg/day (administered as 1 mg on five days per week). Baseline laboratory values were as follows: RF, 280 IU/mL; MMP-3, 108.2 ng/mL; CRP, 0.76 mg/dL; DAS28-CRP, 1.9; serum triglycerides, 184 mg/dL (non-fasting); and aspartate aminotransferase (AST), 29 U/ (Table 1).

**Table 1:** Serial laboratory and clinical parameters during the treatment course Pemafibrate was initiated at month 0 at 0.2 mg/day and increased to 0.4 mg/day at week 12. Triglycerides were measured in non-fasting samples. Tender and swollen joint counts were assessed using the 28-joint count. RF: rheumatoid factor, MMP-3: matrix metalloproteinase-3, CRP: C-reactive protein, DAS28-CRP: 28-joint Disease Activity Score based on CRP, AST: aspartate aminotransferase, ALT: alanine aminotransferase

Variable	Baseline	Month 3	Month 6	Month 12	Month 20	Reference range
RF (IU/mL)	280	190	180	183	166	≤15
MMP-3 (ng/mL)	108.2	58.5	43.9	33.6	42.7	17.3-59.7
CRP (mg/dL)	0.76	0.38	0.93	0.58	0.51	≤0.3
Tender joint count (28 joints)	0	0	0	0	0	-
Swollen joint count (28 joints)	0	0	0	2	0	-
Patient global assessment (mm)	12	9	11	10	11	-
DAS28-CRP	1.9	1.65	1.95	2.19	1.76	-
Triglycerides (mg/dL)	184	71	95	-	88	<175
AST (U/L)	29	40	49	37	25	10-40
Prednisolone (mg/day)	0.7	0.7	0.7	0.33	0	-
Body weight (kg)	71	71	71	71	70	-

Pemafibrate was started at 0.2 mg/day (0.1 mg twice daily) for hypertriglyceridemia and increased to 0.4 mg/day (0.2 mg twice daily) after 12 weeks. Throughout the 20-month observation period, methotrexate, bucillamine, and iguratimod were maintained at fixed doses, so that the observed changes could not be attributed to intensification of conventional antirheumatic therapy. Her body weight remained stable (70 kg at the end of the observation period), and no change in diet or physical activity was reported.

After the initiation of pemafibrate, RF and MMP-3 declined progressively (Figure 1A). The changes described below are reported in temporal relation to the initiation of pemafibrate; no causal inference is intended.

**Figure 1:**
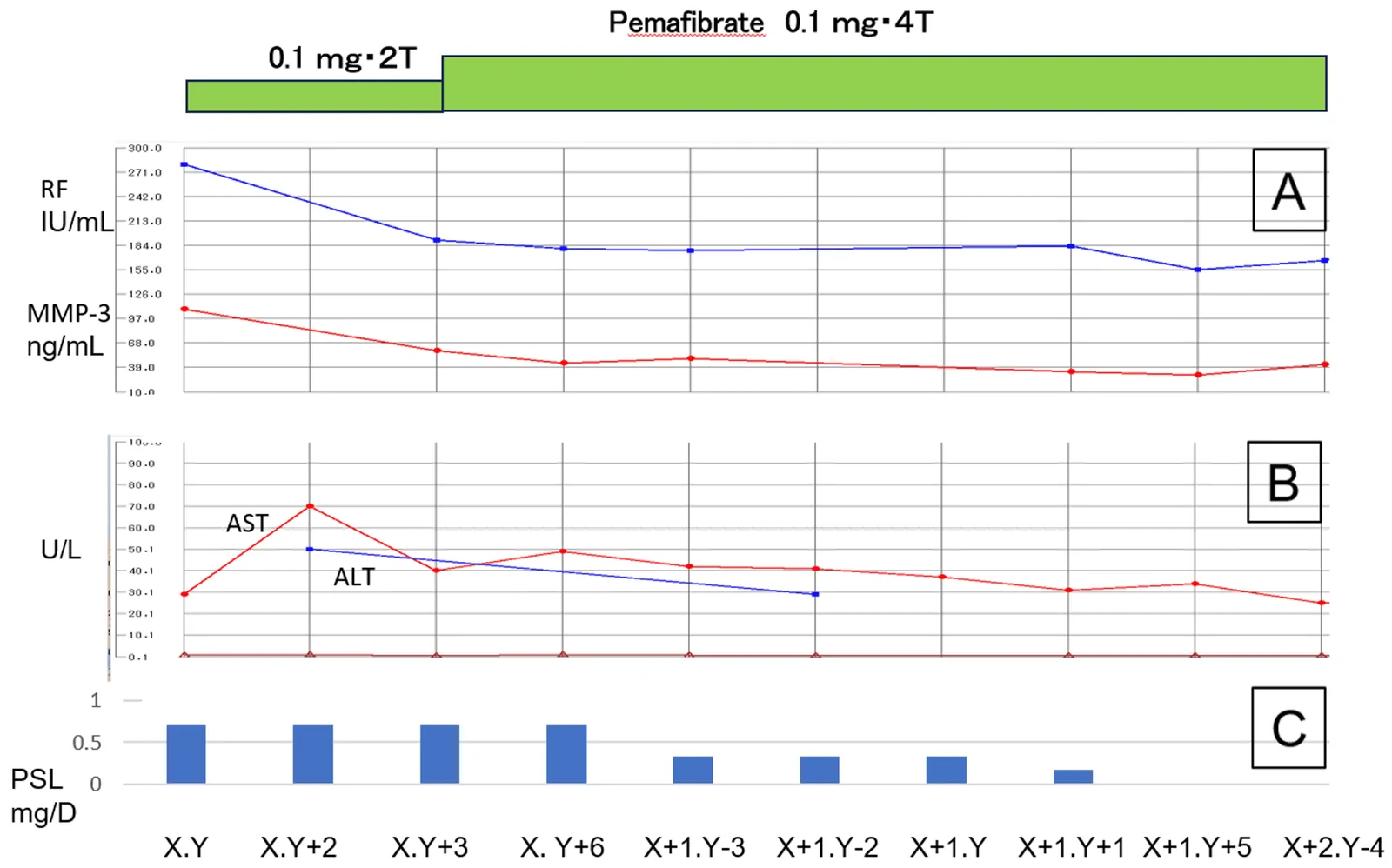
Longitudinal changes in RF and MMP-3 (A), hepatic transaminases (B), and prednisolone dose (C) before and after the initiation of pemafibrate Pemafibrate was started at 0.2 mg/day (0.1 mg twice daily) and increased to 0.4 mg/day (0.2 mg twice daily) after 12 weeks, as indicated by the horizontal bar at the top of Figure 1A. A: RF (blue) and MMP-3 (red), B: AST (blue) and ALT (red), C: daily PSL dose (mg/day) Following pemafibrate initiation, RF and MMP-3 decreased, hepatic transaminases (AST and ALT) showed a transient early increase followed by a return toward baseline, and prednisolone was gradually tapered and eventually discontinued, without any change in the concomitant disease-modifying antirheumatic drug regimen (methotrexate, bucillamine, and iguratimod at fixed doses). RF: rheumatoid factor, MMP-3: matrix metalloproteinase-3, AST: aspartate aminotransferase, ALT: alanine aminotransferase, PSL: prednisolone

Prednisolone was gradually tapered and was discontinued after 17 months without any flare of arthritis requiring intensification of treatment, and DAS28-CRP remained below the remission threshold of 2.3 [5] throughout the observation period (Figure 1C and Figure 2B).

**Figure 2:**
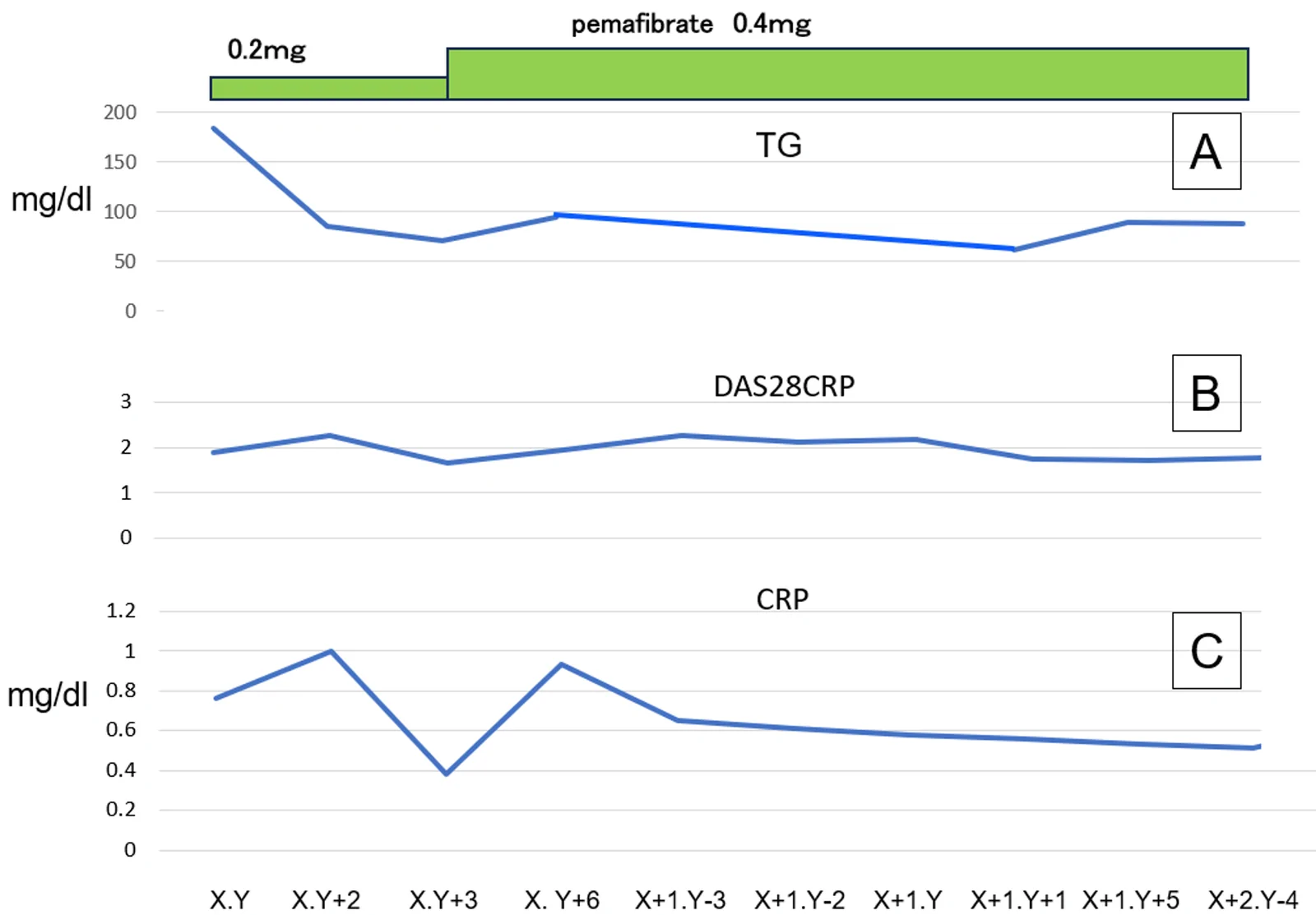
Longitudinal changes in serum triglycerides (A), DAS28-CRP (B), and CRP (C) before and after the initiation of pemafibrate Pemafibrate was started at 0.2 mg/day (0.1 mg twice daily) in X.Y and increased to 0.4 mg/day (0.2 mg twice daily) after 12 weeks, as indicated by the horizontal bar at the top of the figure. A: serum TG (mg/dL; non-fasting samples), B: DAS28-CRP, C: CRP (mg/dL) Following the initiation of pemafibrate, TG decreased and remained stable thereafter. DAS28-CRP remained below the remission threshold of 2.3 throughout the observation period, while CRP peaked during the first six months and thereafter remained around 0.5-0.6 mg/dL. DAS28-CRP: 28-joint Disease Activity Score based on C-reactive protein, CRP: C-reactive protein, TG: triglycerides

The tender joint count (28-joint assessment) was 0 at all 10 assessments. Transient swelling of one joint was noted at month 2, and swelling of one or two joints was noted between months 9 and 12, during prednisolone tapering; these findings resolved without any change in the DMARD regimen or an increase in the prednisolone dose, and no swollen joints were detected at any subsequent assessment, including after prednisolone discontinuation (Table 1).

Serum triglycerides decreased from 184 mg/dL to 88 mg/dL (Figure 2A), consistent with the established lipid-lowering action of pemafibrate in the setting of coexisting MASLD. Hepatic transaminases (AST and alanine aminotransferase (ALT)) showed a transient early increase followed by a return toward baseline (Figure 1B). Pemafibrate was well tolerated: no adverse events attributable to the drug, including myalgia, elevation of creatine kinase, renal dysfunction, or drug-induced liver injury, were observed during the 20-month observation period.

## Discussion

The beneficial effects of pemafibrate have been reported in various preclinical models of inflammatory and fibrotic conditions, including neuroinflammation, myocardial ischemia-reperfusion injury, steatohepatitis-associated cardiomyopathy, atherosclerosis, and peritoneal fibrosis [8-12]. PPARα activation promotes the inactivation of NF-κB, in part through direct interaction with its p65 subunit and through increased expression of IκBα [1]. These findings provide a plausible mechanistic basis by which a PPARα agonist could modulate an NF-κB-driven inflammatory disease such as RA. Although pemafibrate itself has not yet been evaluated in animal models of RA, the anti-arthritic effects of other fibrates have been demonstrated in such models. In transgenic mice overexpressing human apolipoprotein C-III, hypertriglyceridemia exacerbated antigen-induced arthritis, with greater joint swelling, inflammatory infiltration, and cartilage destruction than in non-transgenic controls, and treatment with fenofibrate lowered triglycerides, restored the antioxidant function of high-density lipoprotein, reduced macrophage activation, and significantly ameliorated arthritis severity [13]. This finding is of particular relevance to the present patient, in whom RA coexisted with hypertriglyceridemia. Bezafibrate, a pan-PPAR agonist, significantly attenuated collagen-induced arthritis in rats through PPAR-dependent modulation of inflammatory pathways, although this effect was attributed mainly to PPARγ activation [14]. In addition, fenofibrate exerted protective effects in a complete Freund’s adjuvant-induced model of arthritis in rats [15]. Taken together, these animal data support the concept that PPAR agonism, including PPARα activation, can attenuate experimental arthritis, and they suggest that pemafibrate, a more potent and selective PPARα agonist, may have a similar potential. Nevertheless, because the relative contributions of PPARα and other PPAR subtypes differ among these agents, direct evaluation of pemafibrate in animal models of arthritis is warranted.

Clinical evidence linking fibrates to RA disease activity remains limited but is not without precedent. Okamoto and Kamatani reported a patient in whom the addition of fenofibrate 300 mg/day to methotrexate, bucillamine, and prednisolone was followed by improvement in multiple disease activity parameters, including the pain visual analogue scale, patient’s and physician’s global assessments, the Health Assessment Questionnaire, painful joint count, and CRP [16]. Beyond that single case, two small prospective studies have examined fenofibrate in RA. Goto randomly allocated 44 patients with RA and dyslipidemia to fenofibrate or a statin for six months and recorded the visual analogue scale, CRP, erythrocyte sedimentation rate and, notably, the prednisolone dose [17]. Shirinsky et al. administered fenofibrate 145 mg/day for three months to 27 patients with active RA receiving stable conventional DMARDs and observed significant reductions in DAS28 and in all of its individual components except the tender joint count, in the duration of morning stiffness, and in CRP and interleukin-6, with 60% of patients achieving a good or moderate EULAR response [18]. The rationale for regarding PPARα agonists as candidate disease-modifying agents in RA has been reviewed in detail [19]. The temporal pattern observed in the present case, which involves a different PPARα agonist, is consistent with these earlier clinical observations and extends them to pemafibrate. A single uncontrolled case cannot establish that pemafibrate produced the changes; nevertheless, the resemblance raises the possibility that the anti-inflammatory effect of PPARα agonists is a class-related phenomenon relevant to RA. That the prednisolone dose was among the parameters followed in the comparative study of fenofibrate [17] is of particular interest here, since glucocorticoid withdrawal was one of the principal observations in the present patient.

With regard to dose, the most pronounced decreases in RF and MMP-3 occurred during the initial 12 weeks at 0.2 mg/day, and the subsequent increase to 0.4 mg/day was not followed by a further substantial change in RF. However, MMP-3 continued to decline, reaching its nadir at month 12, and prednisolone was tapered and discontinued only after the dose increase, without any flare requiring intensification of treatment. If the association is genuine, these observations would raise the possibility that the anti-inflammatory effect of pemafibrate had not reached its maximum at 0.2 mg/day. Because the dose was increased for lipid control rather than to assess a dose-response relationship, and because the effects of dose and treatment duration cannot be separated in a single patient, this interpretation remains speculative.

Several limitations should be acknowledged. First, this is a single case, and a causal relationship between pemafibrate and the improvement in RA parameters cannot be established; the natural fluctuation of disease activity and regression toward the mean cannot be excluded. Notably, DAS28-CRP was already within the remission range (1.9) at baseline, so the principal clinical observations were the reduction in inflammatory and disease-related biomarkers and the successful glucocorticoid withdrawal, rather than a change in the composite disease activity score. Consistent with this, the tender joint count was 0 at every assessment and the swollen joint count never exceeded two (Table 1). Moreover, the baseline glucocorticoid dose was already low (prednisolone: 0.7 mg/day); although its complete discontinuation without any flare requiring intensification of DMARD or glucocorticoid therapy is noteworthy, the magnitude of the steroid-sparing effect should not be overstated. Nevertheless, current European Alliance of Associations for Rheumatology (EULAR) recommendations advise that glucocorticoids be tapered and discontinued as rapidly as clinically feasible [20], so the withdrawal of even a low dose is consistent with this treatment goal. Second, the concurrent improvement in triglycerides and liver enzymes indicates a general metabolic benefit, and it is difficult to disentangle a direct antirheumatic effect from indirect effects mediated by improved metabolic and hepatic status. Third, pemafibrate itself has not yet been examined in animal models of arthritis, so mechanistic inference rests on data obtained with other fibrates in experimental arthritis [13-15] and with pemafibrate in other inflammatory conditions [8-12]. Fourth, unlike AST, ALT was not measured continuously throughout the observation period. Although AST may also reflect non-hepatic sources, ALT is more specific to hepatic injury; therefore, serial ALT measurement would have allowed the hepatic effect of pemafibrate to be demonstrated more clearly. ALT monitoring was discontinued after the transaminase levels had stabilized, reflecting a clinical rather than a protocol-based decision.

Disease activity was assessed using the DAS28-CRP rather than the DAS28-ESR. In Japan, health insurance regulations generally allow only one inflammatory marker (either CRP or ESR) to be reimbursed at each assessment, and CRP was selected because it is measured more readily and reproducibly in our institution than ESR. Because CRP had been used consistently both before and throughout the observation period, changing the marker midway would have rendered the serial scores non-comparable. It should be emphasized, however, that the two indices are not interchangeable: DAS28-CRP typically yields values approximately 0.6 points lower than DAS28-ESR in the same patient, so the conventional remission cut-off of 2.6, which was derived from ESR-based data, is too permissive when applied to the CRP-based index. For this reason, remission in the present case was assessed against the stricter DAS28-CRP threshold of 2.3 proposed by Inoue et al. [5]. Clinicians who rely on DAS28-ESR should therefore expect somewhat higher absolute scores than those reported here and should apply the ESR-specific cut-off rather than the threshold used in this report. Where ESR is the more accessible marker, DAS28-ESR remains an entirely appropriate instrument for monitoring disease activity, and nothing in this report argues otherwise.

## Conclusions

In this patient with RA and hypertriglyceridemia, the initiation of pemafibrate was temporally associated with reductions in RF, MMP-3, and CRP, and with the successful withdrawal of low-dose glucocorticoid, while the DMARD regimen remained unchanged. Given that the baseline prednisolone dose was only 0.7 mg/day, this withdrawal should be viewed as a clinically relevant observation rather than as strong evidence of a glucocorticoid-sparing effect. Although causality cannot be established from a single case, the temporal association, the uneventful glucocorticoid withdrawal, and the supporting mechanistic, animal model, and clinical literature suggest that PPARα agonists such as pemafibrate warrant further investigation as potential adjunctive agents in RA, particularly in patients with coexisting dyslipidemia or steatotic liver disease. Controlled studies and, ideally, studies of pemafibrate in animal models of arthritis will be required to confirm and clarify this possible effect.
